# Predicting 28-day all-cause unplanned hospital re-admission of patients with alcohol use disorders: a machine learning approach

**DOI:** 10.1093/alcalc/agaf036

**Published:** 2025-06-23

**Authors:** Jingxiang Zhang, Siyu Qian, Guoxin Su, Chao Deng, David Reid, Barbara Sinclair, Ping Yu

**Affiliations:** Institute of Medical Information, Chinese Academy of Medical Sciences and Peking Union Medical College, Chaoyang District, 3 Yabao street, Beijing 100020, China; Centre for Digital Transformation, School of Computing and Information Technology, Faculty of Engineering and Information Sciences, University of Wollongong, Northfields Ave, Wollongong, New South Wales 2522, Australia; Drug and Alcohol Service, Illawarra Shoalhaven Local Health District, 2 Rawson Street, Wollongong, New South Wales 2520, Australia; School of Computing and Information Technology, Faculty of Engineering and Information Sciences, University of Wollongong, Northfields Ave, Wollongong, New South Wales 2522, Australia; School of Medical, Indigenous and Health Sciences, Faculty of Science, Medicine and Health, University of Wollongong, Northfields Ave, Wollongong, New South Wales 2522, Australia; Drug and Alcohol Service, Illawarra Shoalhaven Local Health District, 2 Rawson Street, Wollongong, New South Wales 2520, Australia; Drug and Alcohol Service, Illawarra Shoalhaven Local Health District, 2 Rawson Street, Wollongong, New South Wales 2520, Australia; Centre for Digital Transformation, School of Computing and Information Technology, Faculty of Engineering and Information Sciences, University of Wollongong, Northfields Ave, Wollongong, New South Wales 2522, Australia

**Keywords:** alcohol use disorder, hospital re-admission, machine learning, Clinical Bio-BERT, long-short term memory

## Abstract

**Introduction:**

Patients with alcohol use disorders have a high hospital re-admission rate, adding to the strain on the healthcare system. To address this issue, this study aimed to predict 28-day unplanned hospital re-admission for these patients.

**Methods:**

From linked de-identified datasets, patients with alcohol use disorders who had hospital re-admissions between 2015 and 2018 were identified. Univariate and multiple logistic regression were conducted to select variables for inclusion in five machine learning models—logistic regression (baseline), random forest, support vector machine, long-short term memory and clinical bio bidirectional encoder representation of transformers (Clinical Bio-BERT)—to predict the 28-day re-admission.

**Results:**

Eight hundred and sixty-nine patients with alcohol use disorders incurred 2254 hospital admissions. Patients aged 45–49 or 70–74 or 75–79 were 4–5 times more likely to be re-admitted than those in other age groups; males were 36% more likely than females; patients who use polysubstance were 3.3 times more likely than otherwise. Patients with “respiratory system disorders” or “hepatobiliary system and pancreas disorders” had 60% higher risk than otherwise. Interaction with emergency department or drug and alcohol service after discharge reduced the risk by 71% and 79%, respectively. The 10-variable Clinical Bio-BERT demonstrated the highest sensitivity (.724).

**Discussion and Conclusions:**

Patients with alcohol use disorders with the following characteristics were more likely to have unplanned re-admissions within 28 days: male, aged 45–49 or 70–74 or 75–79, with “respiratory system disorders” or “hepatobiliary system and pancreas disorders”, or patients who use polysubstance. Interactions with emergency department or drug and alcohol service after discharge had reduced risk of hospital re-admission.

## Teaser

- For patients with alcohol use disorders, being male, aged 45–49 or 70–74 or 75–79, with “respiratory system disorders” or “hepatobiliary system and pancreas disorders”, or patients who use polysubstance are more likely to be re-admitted within 28 days.
- Patients who interact with the emergency department or community-based drug and alcohol service after a hospital discharge have a reduced risk of hospital re-admission within 28 days.
- The Clinical Bio-BERT model is highly sensitivity on predicting re-admissions of patients with alcohol use disorders, in comparison with random forest, support vector machine and long-short term memory.

## Introduction

Alcohol is a widely abused substance that can lead to illnesses, injuries, and fatalities (Johnson et al. 2014, Hobday et al. 2015, Song et al. 2019). According to the US “Healthcare Cost and Utilization Project Statistical Brief”, 19% of hospital re-admissions were caused by alcohol-related diseases in 2013 (Fingar and Washington 2006). Research shows that patients with alcohol use disorders have high hospital re-admission rates (Qian et al. 2019, Hansen et al. 2020, Mogos et al. 2021, Calabrese et al. 2022) and are more likely to have unplanned re-admissions (Gili-Miner et al. 2014). Unplanned re-admissions indicate suboptimal care and are costly (Rashid and Khalil 2011, Chan and Wong 2014, Braet et al. 2015). Therefore, it is of great importance to reduce unplanned hospital re-admissions of patients with alcohol use disorders.

Several studies sought to identify re-admission risk factors for patients with alcohol use disorders so as to reduce unplanned hospital re-admission. These risk factors include multiple comorbidities (Wani et al. 2019), psychiatric comorbidities (Yedlapati and Stewart 2018, Ghosh et al. 2022), prior detoxification history (Ponzer et al. 2002), discharge against medical advice (Yedlapati and Stewart 2018, Hansen et al. 2020), poor income, and low socioeconomic status (Hoy 2017, Yedlapati and Stewart 2018, Wani et al. 2019). Another strategy to reduce re-admission is to develop prediction models, so that preventive interventions can be provided to those at higher risk. However, research on developing prediction models for re-admission of this patient group is scarce.

Machine learning techniques have been widely applied to predict the risk of hospital re-admission (Morel et al. 2020). Hung et al. used support vector machine (SVM) to predict re-admission within 30 days for patients with atrial fibrillation who underwent catheter ablation (Hung et al. 2020). Shang et al. developed and compared machine learning-based methods for predicting re-admission within 30 days for patients with diabetes and found that random forest (RF) model outperformed decision tree and Naive Bayes models (Shang et al. 2021). Long-short term memory (LSTM) network can capture crucial information from electronic medical records that traditional machine learning methods struggle to achieve (Gers and Schmidhuber 2001). Studies have shown that LSTM performs well in predicting hospital mortality (Shi et al. 2020), 30-day unplanned hospital re-admission (Reddy and Delen 2018, Lin et al. 2019, Khekare et al. 2025) and long-term hospital stay (Wang et al. 2020). Recently, Bidirectional Encoder Representation of Transformers (BERT) has been highly successful in clinical text processing. Alsentzer et al. trained Clinical BERT and Clinical Bio-BERT models using all clinical records and discharge-only summaries (Alsentzer et al. 2019). Clinical Bio-BERT was pretrained on all notes from Medical Information Mart for Intensive Care, a database containing electronic health records from ICU patients in the US (Johnson et al. 2016). A recent literature review on current trends in predicting hospital re-admission concludes that overall machine learning models tend to outperform statistical models (Teo et al. 2023). Therefore, this study adopted a machine learning approach for predicting hospital re-admission.

Research has used 30 days and 28 days to measure hospital re-admission. In Australia, the 28-day hospital re-admission has been commonly used as an indicator of hospital medical quality (Li et al. 2015, Considine et al. 2019). Therefore, this study aimed to predict the risk of 28-day all-cause unplanned hospital re-admission of patients with alcohol use disorders in an Australian regional health district using a machine learning approach.

## Methods

This retrospective study took place in the Illawarra Shoalhaven Local Health District (ISLHD), New South Wales, Australia. The district serves a population of around 390 000 residents across rural, regional, and metropolitan areas.

### Datasets

Four de-identified datasets linked by unique patient identifiers were obtained from the Illawarra Health Information Platform managed by the CHRISP (Zhang et al. 2022): emergency department (ED) data (December 2011–January 2019), hospital admitted patient (AP) data (October 2011–January 2019), community-based drug and alcohol service (D&A service) data (November 2014–January 2019) and mental health service data (August 2012–January 2019). The ED and AP datasets classified diagnoses with the Systematic Nomenclature of Medicine Clinical Terms (SNOMED CT) codes and the International Classification of Diseases 10th Revision (ICD-10) codes Australian modification, respectively. Based on discharge diagnoses (see Appendix Table 1), 2519 patients were identified with alcohol use disorders.

### Data preprocessing

Data preprocessing, including data cleaning and transformation to make an analysis-ready dataset for modeling, was performed using R 3.6.1 (Team 2019).

The output variable is re-admission in 28 days, presented with value “1” for yes (i.e. re-admitted within 28 days of discharge, excluding the 28th day) or “0” for no (i.e. re-admitted after 28 days or not re-admitted after discharge). The time interval between an index admission and a re-admission was calculated to determine whether the re-admission was within or outside 28 days. Index admission is not a particular admission, but the previous admission before the next admission for a patient. We define that:

- an index admission (i) can be a planned or unplanned admission and (ii) the patient must be discharged alive.
- a re-admission (i) must be the immediate unplanned admission following the index admission, (ii) cannot be a planned admission, (iii) could be for any reason, and (iv) the patient could be alive or dead at discharge.

Forty-two potential input variables are presented in Table 1. Most variables (e.g. sex, Aboriginality) were categorical variables with dichotomous values, recorded as “1” for yes and “0” for no. Eleven variables (e.g. marital status) had more than two categories. Polysubstance use is one of the potential input variables. The World Health Organization “Lexicon of alcohol and drug terms” defines polysubstance use as “the use of more than one drug or type of drug by an individual” (Bradbury 1995). This definition was used to identify patients with polysubstance use and the identification process has been described in (Zhang et al. 2022, Zhang et al. 2024). The categorical variable “complications and comorbidities” has five levels of severity (i.e. no effect, minor, moderate, severe, and catastrophic). According to the data dictionary, this variable is “a measure of the cumulative effect of a patient’s complications and comorbidities and is calculated for each episode”. As one hospital admission may contain more than one episode, we used the severity level recorded in the last episode (i.e. at the end of the admission), as a patient’s condition at discharge may impact on how soon the patient would return to hospital.

**Table 1:** The 42 variables included in the analysis

**No.**	**Variable**	**Description**
1	Age group	The age group of a patient at the date of their first admission to the hospital, spanning from 18–24, 25–29,30–34, ..., 80–84, 85+.
2	Aboriginality	A patient is Aboriginal or Torres Strait Islander.
3	Marital status	Never married/widowed/divorced/separated/married/unknown
4	Sex	Female/male
5	Mode of separation	The status of the person at departure from the health facility. This includes discharge by hospital, discharge at own risk, and other.
6	Intensive care unit (ICU) status	The type of ICU service, if any, a patient received during an episode of hospital care. Two categories: No ICU admission/ICU admission.
7	Major diagnostic category	Twenty-one mutually exclusive diagnostic categories, corresponding to a single body system or cause, broadly reflecting the specialty that provides care. This includes diseases and disorders of the nervous system; the eye; the ear, nose, mouth and throat; the respiratory system; the circulatory system; the digestive system; the hepatobiliary system and pancreas; the musculoskeletal system and connective tissue; the skin, subcutaneous tissue and breast, endocrine, nutritional and metabolic diseases and disorders; the kidney and urinary tract; the male reproductive system; the female reproductive system; the blood and blood forming organs and immunological disorders; neoplastic disorders (hematological and solid neoplasms); infectious and parasitic diseases; mental diseases and disorders; alcohol/drug use and alcohol/drug induced organic mental disorders; injuries; poisoning and toxic effect of drugs; burns; factors influencing health status and other contacts with health services.
8	ED status	A flag that indicates if a patient has been treated within the ED during an episode of hospital care.
9	Patient type	Grouping patient episodes. This includes acute, psychiatric, sub-acute, and non-acute.
10	Surgery indicator	An indication whether, at separation, the episode of care was defined as surgical, medical, or procedural.
11	Patients who use polysubstance	A patient with alcohol use disorders and a diagnosis of another class of substance, e.g. opioids, stimulants.
12	Emergency status	For each episode of ED care, report whether or not the patient was admitted for care or treatment which, in the opinion of the treating clinician, was necessary within 24 h.
13	Referred to on separation	Service to which the patient was referred to on separation from this episode of admitted care. The categories include not referred, private medical practitioner other than psychiatric or other.
14	Source of referral	The source from which the person was transferred/referred for an episode of AP care. This includes ED, hospital in same health service, medical practitioner other than private psychiatric practice, or other.
15	Complications and comorbidities	This is a measure of the cumulative effect of a patient’s complications and comorbidities and is calculated for each episode. The calculation is complex and has been designed to prevent similar conditions from being counted more than once. The value is the result returned by the grouping process on loading episode records to the Health Information Exchange according to the code set version of diagnosis-related groups designated as “current”.
16	Alcohol-related primary diagnosis	Whether the primary diagnosis is alcohol-related, e.g. alcoholic myopathy.
17	Primary specialty code	The primary specialty in an admission, e.g. general surgery.
18	ED visit	Whether the patient has been to the ED between the index hospital admission and the unplanned hospital re-admission.
19	D&A Service interaction	Whether the patient has any interaction with the D&A service between the index hospital admission and the unplanned hospital re-admission.
20	Discharge diagnosis 1	Certain infectious and parasitic diseases
21	Discharge diagnosis 2	Neoplasms
22	Discharge diagnosis 3	Diseases of the blood and blood-forming organs and certain disorders involving the immune mechanism.
23	Discharge diagnosis 4	Endocrine, nutritional, and metabolic diseases
24	Discharge diagnosis 5	Mental, behavioral, and neurodevelopmental disorders
25	Discharge diagnosis 6	Diseases of the nervous system
26	Discharge diagnosis 7	Diseases of the eye and adnexa
27	Discharge diagnosis 8	Diseases of the circulatory system
28	Discharge diagnosis 9	Diseases of the respiratory system
29	Discharge diagnosis 10	Diseases of the digestive system
30	Discharge diagnosis 11	Diseases of the skin and subcutaneous tissue
31	Discharge diagnosis 12	Diseases of the musculoskeletal system and connective tissue
32	Discharge diagnosis 13	Diseases of the genitourinary system
33	Discharge diagnosis 14	Symptoms, signs, and abnormal clinical and laboratory findings, not elsewhere classified.
34	Discharge diagnosis 15	Injury, poisoning, and certain other consequences of external causes.
35	Discharge diagnosis 16	Codes for special purposes
36	Discharge diagnosis 17	External causes of morbidity
37	Discharge diagnosis 18	Factors influencing health status and contact with health services.
38	Length of stay in index admission	The number of days between the hospital admission date and discharge date.
39	Days in psych unit	The number of days the person was accommodated in a designated psychiatric unit.
40	Hours on mech vent num	Hours on mechanical ventilation with certain number.
41	Number of specialties	The number of specialty category in a hospital admission.
42	Number of diagnoses	Number of diagnoses in a hospital admission.

As the four datasets had different time periods, we narrowed down the data to the four natural years 2015–18, to investigate the variable “D&A service interaction”. As a result, 1404 patients were included; of them, 535 had interactions with the mental health service. During the development of the prediction models, we found that excluding patients who had interactions with the mental health service would improve the homogeneity of the data, leading to improved model performance. Therefore, these patients were excluded. As a result, the final total number of patients included in the analysis was 869 patients.

### Selection of factors associated with hospital re-admission

Risk factors associated with hospital re-admission were selected from the 42 potential variables. Analysis was conducted using IBM SPSS for Windows version 26. First, we conducted univariate binomial logistic regression (LR) to identify factors associated with 28-day re-admission (Havens et al. 2015, Direkvand-Moghadam et al. 2016, Crosetti et al. 2019, Xu et al. 2021) and excluded the factors not significantly associated with re-admission (Havens et al. 2015, Xu et al. 2021). The *P*-value was set to .10 as suggested by Wang (Wang et al. 2017). As a result, 24 variables were included in the next step of multivariate LR to identify independent associations between the selected variables and the 28-day re-admission, with *P* < .05 as the sign of significant association (see Appendix Table 2). As a result, 10 variables were included in the prediction models: age group, sex, mode of separation, major diagnostic category, patients who use polysubstance, source of referral, complications and comorbidities, alcohol-related primary diagnosis, ED visit, and interactions with D&A service.

### Training and testing machine learning models to predict 28-day all-cause unplanned hospital re-admission

We experimented with five models, LR (adopted as the baseline), RF, SVM, LSTM, and Clinical Bio-BERT, to build prediction models. We included 10 independent variables selected above and all 42 independent variables in the five models for comparison. To validate the models, we applied the 10-fold cross-validation method to the RF and SVM models (Lee et al. 2020). For LSTM and Clinical Bio-BERT, we split the data into two portions—70% for training and 30% for testing, as Reddy et al suggested (Reddy and Delen 2018).

### Model performance measurement

Key model performance measurement metrics—area under the curve (AUC), specificity and sensitivity under the best threshold and accuracy—were calculated for the five experimental models (Hung et al. 2020). The AUC is a summary measure of the performance. Accuracy refers to the overall correctness of the prediction. Specificity refers to the percentage of our correct predictions for all patients who did not return to the hospital within 28 days. Sensitivity refers to the percentage of our correct predictions for all patients who returned to the hospital within 28 days.

## Results

### Population characteristics

Eight hundred and sixty-nine patients and their hospital admissions were included in the analysis. They incurred 2254 admissions to nine hospitals in ISLHD (see Appendix Table 3). Of these patients, 75% (650) were males, 9% (81) were Aboriginal, and 7% (61) were polysubstance users. Of these admissions, 80% (1796) were incurred by males, 10% (218) by Aboriginals and 3% (64) by patients who use polysubstance. Each patient was admitted to a hospital .65 times per year on average [95% Confidence Interval (CI): 0.60, 0.70] and the maximum number of admissions was 8.75 times (one patient) per year.

The mean LOS at the index admission was 7.00 h [Standard Deviation (SD): 9.89 h, 95% CI: 6.62, 7.44], the median was 4.00 h [Interquartile Range (IQR): 2.00, 8.00 h]. Of all the admissions, 80% (1809) were referred from ED and 20% (445) were referred from another hospital in ISLHD. In 89% (2009) of the admissions, patients were discharged by the hospital. In 6% (132) of the admissions, patients were discharged at their own risk.

### Risk factors for 28-day all-cause unplanned hospital re-admission

Ten variables were associated with 28-day all-cause unplanned hospital re-admission (Table 2). Patients aged 45–49, 70–74, and 75–79 were over 4–5 times more likely to be re-admitted in 28 days than those aged 18–24 [Odds Ratio (OR) = 4.650, 95% CI = 1.028, 21.036, *P =* .046; OR = 4.455, 95% CI = 1.005, 19.746, *P* = .049; OR = 5.412, 95% CI = 1.207, 24.272, *P =* .027]. In addition, males had a 36% higher risk of being re-admitted to hospital in 28 days than females (OR = .636, 95% CI = .488, .829, *P =* .001). Patients who use polysubstance were over 3 times more likely to be re-admitted in 28 days than those not (OR = 3.273, 95% CI = 1.790, 5.983, *P* < .001).

**Table 2:** Risk factors for 28-day all-cause unplanned hospital re-admission

**Variable**	**Number of admissions (*n* = 2254)**	**OR** * **(95% CI)**	***P-value* in multiple LR**
**Age group**			.030
18–24	28 (1%)	1 [reference]	
25–29	23 (1%)	0.593 (.049, 7.131)	.681
30–34	39 (2%)	2.701 (.483, 15.098)	.258
35–39	67 (3%)	2.020 (.390, 1.450)	.402
40–44	108 (5%)	2.294 (.483, 1.902)	.296
45–49	173 (8%)	4.650 (1.028, 21.036)	.046
50–54	297 (13%)	4.282 (.965, 18.998)	.056
55–59	283 (13%)	3.534 (.796, 15.688)	.097
60–64	314 (14%)	3.900 (.880, 17.273)	.073
65–69	271 (12%)	3.348 (.751, 14.934)	.113
70–74	308 (14%)	4.455 (1.005, 19.746)	.049
75–79	180 (8%)	5.412 (1.207, 24.272)	.027
80–84	92 (4%)	3.820 (.815, 17.903)	.089
85+	71 (3%)	2.563 (.531, 12.383)	.241
**Sex**			
Male	1796 (80%)	1 [reference]	
Female	458 (20%)	0.636 (.488, .829)	.001
**Mode of separation**			<.001
Discharge by hospital	2009 (89%)	1 [reference]	
Discharge at own risk	132 (6%)	1.464 (.970, 2.209)	.069
Other**	113 (5%)	3.981 (2.257, 7.020)	<.001
**Major diagnostic category**			.031
Diseases and disorders of the nervous system	223 (10%)	1 [reference]	
Diseases and disorders of the eye	8 (<1%)	0.879 (.166, 4.671)	.880
Diseases and disorders of the ear, nose, mouth, and throat	36 (2%)	1.352 (.579, 3.156)	.485
Diseases and disorders of the respiratory system	225 (10%)	1.578 (1.017, 2.448)	.042
Diseases and disorders of the circulatory system	231 (10%)	1.415 (.917, 2.183)	.116
Diseases and disorders of the digestive system	271 (12%)	1.286 (.838, 1.974)	.249
Diseases and disorders of the hepatobiliary system and pancreas	498 (22%)	1.564 (1.008, 2.428)	.046
Diseases and disorders of the musculoskeletal system and connective tissue	124 (6%)	0.694 (.394, 1.222)	.206
Diseases and disorders of the skin, subcutaneous tissue and breast	61 (3%)	0.600 (.283, 1.273)	.183
Endocrine, nutritional, and metabolic diseases and disorders	82 (4%)	1.073 (.590, 1.952)	.817
Diseases and disorders of the kidney and urinary tract	69 (3%)	1.259 (.669, 2.369)	.476
Diseases and disorders of the male reproductive system	5 (<1%)	Nil	Nil
Diseases and disorders of the female reproductive system	4 (<1%)	Nil	Nil
Diseases and disorders of the blood and blood forming organs, and immunological disorders	43 (2%)	0.623 (.270, 1.438)	.268
Neoplastic disorders (hematological and solid neoplasms)	4 (<1%)	Nil	Nil
Infectious and parasitic diseases	65 (3%)	0.955 (.491, 1.860)	.893
Mental diseases and disorders	11 (1%)	1.477 (.317, 6.886)	.620
Alcohol/drug use and alcohol/drug induced organic mental disorders	119 (5%)	2.053 (.995, 4.235)	.052
Injuries, poisoning, and toxic effect of drugs	78 (4%)	0.851 (.452, 1.603)	.618
Burns	3 (<1%)	Nil	Nil
Factors influencing health status and other contacts with health services	94 (4%)	2.387 (1.386, 4.111)	.002
**Patients who use polysubstance**			
No	2190 (97%)	1 [reference]	
Yes	64 (3%)	3.273 (1.790, 5.983)	<.001
**Source of referral**			.018
Emergency Department	1809 (80%)	1 [reference]	
Hospital in same Health Service	335 (15%)	0.684 (.500, .936)	.017
Medical Practitioner other than Private Psychiatric Practice	91 (4%)	1.597 (.984, 2.593)	.058
Other***	19 (1%)	0.995 (.369, 2.683)	.992
**Complications and comorbidities**			.002
No complications and comorbidities effect	376 (17%)	1 [reference]	
Minor complications and comorbidities	31 (1%)	2.163 (.918, 5.095)	.078
Moderate complications and comorbidities	459 (20%)	0.686 (.478, .986)	.041
Severe complications and comorbidities	559 (25%)	0.952 (.675, 1.342)	.777
Catastrophic complications and comorbidities	829 (37%)	1.268 (.870, 1.847)	.216
**Alcohol-related primary diagnosis**			
No	1777 (79%)	1 [reference]	
Yes	477 (21%)	0.513 (.340, .775)	.002
**ED visit between the index and re-admission**			
No	166 (7%)	1 [reference]	
Yes	2088 (93%)	0.294 (.197, .439)	<.001
**Interaction with D&A Service between the index and re-admission**			
No	2083 (92%)	1 [reference]	
Yes	171 (8%)	0.215 (.120, .386)	<.001

*P* < .05 means that in the currently fitted model, the included variable has a statistically significant OR value.

^a^Odds ratio (OR): measure of association between an exposure and an outcome; CI: confidence interval.

^b^Other: include transfer to nursing home, transfer to other accommodation, discharge on leave, transfer to palliative care unit / hospice.

^c^Other: include Community Health, Outpatients, Other Hospital/Day Procedure Centre, Nursing Home/ Residential Aged Care Facility, Other Agency, Private Psychiatric Practice, Law Enforcement Agency, Mental Health Crisis Team, Self, Unknown.

Patients with respiratory system disorders (OR = 1.578, 95% CI = 1.017, 2.448, *P* = .042) and hepatobiliary system and pancreas disorders (OR = 1.564, 95% CI = 1.008, 2.428, *P* = .046) were about 1.6 times more likely to be re-admitted in 28 days than those with other diseases.

Patients referred from hospitals in the same health service had a 32% lower 28-day re-admission risk than patients referred from the ED (OR = .684, 95% CI = .500, .936, *P* = .017). Patients with moderate complications and comorbidities had a 31% lower risk of 28-day re-admission compared to patients with no complications and comorbidities (OR = .686, 95% CI = .478, .986, *P* = .041). In addition, patients with an alcohol-related primary diagnosis had a 49% reduced risk of being re-admitted within 28 days compared to those without (OR = .513, 95% CI = .340, .775, *P =* .002). Furthermore, patients who had interactions with ED or D&A service between the index admission and the unplanned re-admission had 71% and 79% reduced risk of being re-admitted within 28 days than those without these interactions, respectively (OR = .294, 95% CI = .197, .439, *P <* .001; OR = .215, 95% CI = .120, .386, *P* < .001).

### Machine learning model performance for predicting 28-day all-cause unplanned hospital re-admission

Table 3 presents the performance of the machine learning model, measured by metrics including accuracy, specificity, and sensitivity in the best threshold, and AUC with 95% CI. Appendix Table 4 presents the receiver operating characteristic curve of the models. Ten-variable LSTM (risk factors identified above) had the highest accuracy (.814). In terms of AUC, the 42-variable Clinical Bio-BERT (.753, 95% CI = .687, .808) performed the best. In addition, sensitivity in Clinical Bio-BERT is higher than that in other models.

**Table 3:** Machine learning model performance metrics

**No. variables**	**Model**	**Accuracy**	**Specificity**	**Sensitivity**	**AUC (95% CI)**
**10**	**LR**	0.730	0.555	0.717	0.687 (.663, .711)
	**RF**	0.725	0.679	0.539	0.657 (.632, .682)
	**SVM**	0.695	0.768	0.465	0.657 (.632, .682)
	**LSTM**	**0.814**	0.841	0.609	**0.749 (.686**, **.814)**
	**Clinical Bio-BERT**	0.728	0.730	0.724	0.744 (.685, .809)
**42**	**LR**	0.664	0.710	0.548	0.680 (.656, .705)
	**RF**	0.625	0.722	0.564	0.694 (.669, .718)
	**SVM**	0.669	0.713	0.555	0.680 (.656, .704)
	**LSTM**	**0.756**	0.778	0.696	0.718 (.657, .777)
	**Clinical Bio-BERT**	0.731	0.739	0.716	**0.753 (.687**, **.808)**

LR, logistic regression; RF, random forest; SVM, support vector machine; LSTM, long-short term memory; BERT, Bidirectional Encoder Representation of Transformers

**Figure 1:**
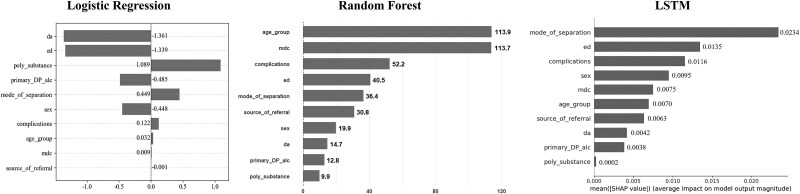
Weight of each factor.

Figure 1 shows the weights of various factors determined by the multiple LR model, RF model and LSTM model.

## Discussion

This study identified 10 risk factors for 28-day all-cause unplanned hospital re-admission of patients with alcohol use disorders: age group, sex, mode of separation, major diagnostic category, polysubstance use, source of referral, complications and comorbidities, alcohol-related primary diagnosis, visit to ED, and interactions with D&A service. The major insight from the research is that (i) postdischarge interactions with ED or community-based D&A service had reduced risk of 28-day all-cause unplanned hospital re-admission; (ii) Certain patient characteristics impact their risk of hospital re-admission; and (iii) Clinical Bio-BERT can be the best model for prediction of hospital re-admission for patients with alcohol use disorders.

### Postdischarge interactions with emergency department or drug & alcohol service can reduce the risk of 28-day all-cause unplanned re-admission

Our findings show that postdischarge interactions with ED or D&A service had 71% and 79% lower risk of hospital re-admission than those who did not have these interactions, respectively. This is consistent with the previous findings that postdischarge engagement with health services reduces risk of hospital re-admission for patients with chronic conditions (Jackson et al. 2015, Gao et al. 2018). Gao et al. found that an enhanced postdischarge engagement with health service providers reduced 30-day re-admissions by 33% for patients with alcohol use disorders (Gao et al. 2018). They also found that patients with at least one postdischarge contact had lower re-admission rates than unengaged patients and increasing postdischarge engagement frequency further decreased re-admission likelihood. Further research can explore the frequency of interactions with ED or D&A service is correlated with re-admissions in our patient group.

### The impact of patient characteristics

A previous study found that male patients were 16% more likely to be re-admitted (Yedlapati and Stewart 2018), we found a higher likelihood of 36% for male patients to be re-admitted into hospitals than female patients. Compared to previous studies showing higher re-admission risk in patients aged 45–64 (Wani et al. 2019) or over 65 years (Considine et al. 2019), our study found that patients aged 45–49 or 70–74, or 75–79 had the higher risk, possibly due to multimorbidities.

Our study found that the mode of separation was a risk factor for 28-day re-admissions. Specifically, patients separated with “other” mode (e.g. transfer to a nursing home, transfer to other accommodation, discharge on leave, transfer to the palliative care unit/hospice) were nearly 4 times more likely to re-admit into hospital than those who were discharged by hospital. Because these modes of separation were too infrequently represented in the study’s data to assess their individual impact on re-admission, further research is necessary to validate the role of each mode in re-admission.

This study found that patients with the major diagnostic categories of diseases and disorders of the respiratory system (OR 1.578), or the hepatobiliary system and pancreas (OR 1.564) were more likely to be re-admitted. Numerous studies have demonstrated an association between alcohol consumption and these two diseases. High alcohol consumption was associated with the risk of several respiratory diseases, such as adult respiratory distress syndrome, and acute lung injury (Boé et al. 2009, Simou et al. 2017). Furthermore, patients with alcohol use disorders have an impaired immune response, causing them to be more susceptible to lung infections (Simet and Sisson 2015). Chronic alcohol abuse can lead to pancreatic damage (Masamune 2015, Pezzilli 2015) and liver disease (Fuster and Samet 2018, Seitz et al. 2018). In addition, there might be a close relationship between pancreatic and liver damage in heavy drinkers (Toskes 2009, Clemens and Mahan 2010).

Patients who use polysubstance (more than one substance in addition to alcohol) had a much higher risk of re-admission than patients with only alcohol use disorders (OR 3.273). Stinson et al.’s study of comorbid alcohol and substance use disorders in the US found that comorbid users were likely to have higher severity of alcohol and/or drug use disorders, which increased their rates of help-seeking (Stinson et al. 2006). Arning explored factors that may predict re-admission to the hospital after detoxification and found that patients who use polysubstance were admitted to the hospital more often than single substance users (Arning 2017). However, further research is needed to examine whether the increased rate of re-admission for patients who use polysubstance is due to the severity of the substance use disorders, comorbidities, or other factors.

Complications and comorbidities are risk factors for re-admission of patients with alcohol use disorders in many studies (Yedlapati and Stewart 2018, Wani et al. 2019, Ghosh et al. 2022). Results of Wani et al.’s study of risk factors for hospital re-admission in patients with alcohol use disorders showed that patients with unrelated comorbidities were more likely to be re-admitted to the hospital within 30 days compared to those without unrelated comorbidities (Wani et al. 2019). However, our study found that patients with moderate complications and comorbidities had a lower risk of re-admission than patients with no complications and comorbidities (OR .686). This contrary finding might be related to how the severity level in the variable “complications and comorbidities” was calculated and classified to measure the cumulative effect; however, the classification was not conducted by the study team, but was provided in the de-identified dataset. Further research into how the cumulative effect of complications and comorbidities would impact hospital re-admission should be conducted.

In addition, this study found that patients referred from hospitals within the same health service (OR .684) had a lower 28-day re-admission risk compared with patients from other referral sources. In addition, patients with an alcohol-related primary diagnosis (OR .513) had a lower risk of re-admission. A possible explanation for this was that hospitals have access to the health records of patients referred from hospitals in the same health service and can provide more targeted services to patients, therefore reducing the risk of re-admission. A study by Singal et al. found that patients hospitalized for alcohol-related cirrhosis who were discharged with a diagnosis of alcohol use disorder had a 30-day lower risk of re-admission (Singal et al. 2022). However, our study considered all-cause re-admissions in patients with alcohol use disorders, and further research was needed to examine the relationship between admissions for alcohol use disorders and admissions for other reasons.

### Comparison of the machine learning models

We found that 10-variable LSTM achieved the highest comprehensive performance with an AUC of .749 (95% CI: .686, .814) and an overall prediction accuracy of .814. The LSTM can capture long-term dependencies between longitudinal clinical events, considering patients’ historical clinical information when predicting future events. The most important value driver for healthcare delivery systems in this study is the ability to accurately predict which patients are at high risk of unplanned hospital re-admission. This is important for community-based health services to implement early interventions before a patient’s health deteriorates to the extent that requires hospitalization. In practice, high sensitivity was more important in screening and identifying patients at higher risk of re-admission (Lin et al. 2019). Therefore, the 10-variable Clinical Bio-BERT is the optimal model for the prediction of hospital re-admission because it had the highest sensitivity among all models.

### Limitation

Research has shown that receiving an addiction consultation during hospitalization was associated with a reduced 30-day re-admission rate (Wakeman et al. 2021). The D&A service in our study not only provides a range of community-based services and programs but also hospital-based D&A consultation liaison (CL) service. In the CL service, specially trained nurses and doctors visit patients in hospitals, provide advice and education on the management of substance use-related conditions, and refer patients for ongoing support with community-based services (Zhang et al. 2022). However, data about the D&A CL services were not available in the de-identified linked datasets used in this study, thus were not included in the analysis. Future studies need to investigate the effect of CL service on linking patients with the D&A service and reducing risk of hospital re-admission.

Patients who had interactions with the mental health service were excluded from the data. Although this may reduce the representativeness of the data, the model performance was improved, indicating two distinct sub-groups within the patients with alcohol use disorders, i.e. those who had interactions with the mental health service and those who did not. Further research is required to investigate risk factors for hospital re-admission for patients with dual pathology.

The data dictionary’s lack of clear definitions of the “complications and comorbidities” levels (i.e. minor, moderate, severe and catastrophic) limits the interpretability of this variable. Cautions should be taken when interpreting this variable.

This study did not incorporate social determinants of health variables such as socioeconomic status, education level, and housing instability because these variables were unavailable in the de-identified datasets. Future research should consider integrating these variables to enhance the model’s predictive accuracy and applicability in real-world settings and provide a more comprehensive assessment of the re-admission risks for this patient group.

Studies showed that inpatient mortality rate and re-admission rates have an inverse relationship (Vaduganathan et al. 2013), i.e. hospitals with low mortality rates tend to have high re-admission rates (Gorodeski et al. 2010, Parina et al. 2015). Glance et al.’s study conducted in the United States also showed a significant difference in hospital performance measured by re-admission rate with a composite indicator that includes or excludes mortality (Glance et al. 2017). As patient deaths occurred on the last admission, which was not counted by our research method, we did not consider the impact of hospital mortality in calculating re-admission rate.

Due to data acquisition restrictions, we could only train and test models using data from a single health district. This lack of external validation may limit the generalizability of our findings across different regions and healthcare systems. In future research, collaborations with teams from other areas or accessing datasets from diverse healthcare settings could be explored to conduct comprehensive external validation, thereby enhancing the models’ applicability in broader contexts.

Compared to the experimental results of Emily et al. (accuracy: 83%) (Alsentzer et al. 2019), the accuracy of Clinical Bio-BERT in our study was poor. This may be because our study solely consists of structured data and incorporating structured data into BERT may constrain the model’s capacity to comprehend and represent structured information. In fact, Bio-BERT is a large language model and has strong capability in processing unstructured data. In the further, maybe unstructured data can also be included in Clinical Bio-BERT to improve model performance in hospital re-admission prediction.

## Conclusions

This study found that patients with the following characteristics were more likely to be re-admitted to hospital within 28 days: male, aged 45–49 or 70–74 or 75–79, major diagnostic categories of “respiratory system disorders”, or “hepatobiliary system and pancreas disorders”, or patients who use polysubstance. Interactions with the ED or community-based D&A service after discharge had reduced the risk of hospital re-admission. The 10-variable Clinical Bio-BERT provided the highest sensitivity among all models, which is the best machine learning model to predict 28-day all-cause unplanned hospital re-admission for patients with alcohol use disorders in this study.

## Supplementary Material

Appendix_agaf036

## Data Availability

The data that support the findings of this study are available from the corresponding author upon reasonable request.

## Notes

*Declaration of competing interest:* The authors declare that they have no known competing financial interests or personal relationships that could have appeared to influence the work reported in this paper..

## Statements

This paper contains original material, not submitted, in press, or published elsewhere in any form.

Each author has contributed significantly to the work and agrees to the submission.
