# Supplementary material for: Predicting 28-day all-cause unplanned hospital re-admission of patients with alcohol use disorders: a machine learning approach
Source: Alcohol Alcohol. 2025 Jun 23;60(4):agaf036. doi: 10.1093/alcalc/agaf036 (PMC12205985; doi:10.1093/alcalc/agaf036)
Supplement: Appendix_agaf036 [file appendix_agaf036.docx]

Appendix A. Supplementary material

**Appendix Table I. SNOMED CT codes and ICD-10 AM codes used to identify people with alcohol use disorders.**

| **No.** | **SNOMED CT Code** | **SNOMED Label** |
| --- | --- | --- |
| 1 | 191802004 | Acute alcoholic intoxication in alcoholism |
| 2 | 9953008 | Acute alcoholic liver disease |
| 3 | 183486001 | Admitted to alcohol detoxification centre (finding) |
| 4 | 15167005 | Alcohol abuse (disorder) |
| 5 | 66590003 | Alcohol dependence |
| 6 | 8635005 | Alcohol withdrawal delirium (disorder) |
| 7 | 191476005 | Alcohol withdrawal hallucinosis |
| 8 | 191480000 | Alcohol withdrawal syndrome |
| 9 | 308742005 | Alcohol withdrawal-induced convulsion |
| 10 | 420054005 | Alcoholic cirrhosis (disorder) |
| 11 | 50325005 | Alcoholic fatty liver |
| 12 | 2043009 | Alcoholic gastritis (disorder) |
| 13 | 235881000 | Alcoholic hepatic failure |
| 14 | 235875008 | Alcoholic hepatitis (disorder) |
| 15 | 41309000 | Alcoholic liver damage (disorder) |
| 16 | 7916009 | Alcoholic polyneuropathy |
| 17 | 235942001 | Alcohol-induced acute pancreatitis |
| 18 | 235952002 | Alcohol-induced chronic pancreatitis (disorder) |
| 19 | 361272001 | Cerebellar ataxia due to alcoholism |
| 20 | 307757001 | Chronic alcoholic hepatitis |
| 21 | 191804003 | Continuous acute alcoholic intoxication in alcoholism |
| 22 | 284591009 | Persistent alcohol abuse |
| 23 | 228281002 | Problem drinker (finding) |
| 24 | 29212009 | Alcohol-induced organic mental disorder (disorder) |
| 25 | 42344001 | Alcohol-induced psychosis (disorder) |
| 26 | 7200002 | Alcoholism |
| 27 | 300992002 | Alcohol-induced cerebellar ataxia |
| 28 | 7052005 | Alcohol hallucinosis (disorder) |
|  | **ICD-10-AM Code** | **ICD-10-AM Label** |
| 29 | E51.2 | Wernicke encephalopathy |
| 30 | F10 | Mental and behavioural disorders due to use of alcohol |
| 31 | G31.2 | Degeneration of nervous system due to alcohol |
| 32 | G62.1 | Alcoholic polyneuropathy |
| 33 | G72.1 | Alcoholic myopathy |
| 34 | I42.6 | Alcoholic cardiomyopathy |
| 35 | K29.2 | Alcoholic gastritis |
| 36 | K70.0 | Alcoholic fatty liver |
| 37 | K70.1 | Alcoholic hepatitis |
| 38 | K70.2 | Alcoholic fibrosis and sclerosis of liver |
| 39 | K70.3 | Alcoholic cirrhosis of liver |
| 40 | K70.4 | Alcoholic hepatic failure |
| 41 | K70.9 | Alcoholic liver disease, unspecified |
| 42 | K85.2 | Alcohol-induced acute pancreatitis |
| 43 | K86.0 | Alcohol-induced chronic pancreatitis |
| 44 | Z50.2 | Alcohol rehabilitation |
| 45 | Z71.4 | Counselling and surveillance for alcohol use disorder |
| 46 | Z86.41 | Personal history of alcohol use disorder |

SNOMED CT^f^: Systematized Nomenclature of Medicine Clinical Terms. ICD-10-AM^g^: International Statistical Classification of Diseases and Related Health Problems, Tenth Revision, Australian Modification

**Appendix Table II. Selection of independent variables**

| No. | Independent variables | Univariate binomial logistic regression | Multivariate binomial logistic regression | Prediction models |
| --- | --- | --- | --- | --- |
| 1 | Age group | <0.001 | 0.030 | included |
| 2 | Aboriginality | 0.318 | excluded | excluded |
| 3 | Marital status | 0.186 | excluded | excluded |
| 4 | Sex | <0.001 | 0.001 | included |
| 5 | Mode of separation | <0.001 | <0.001 | included |
| 6 | Intensive care unit (ICU) status | 0.338 | excluded | excluded |
| 7 | Major diagnostic category | <0.001 | 0.031 | included |
| 8 | ED status | 0.221 | excluded | excluded |
| 9 | Patient type | 0.620 | excluded | excluded |
| 10 | Surgery indicator | 0.421 | excluded | excluded |
| 11 | Polysubstance user | 0.020 | <0.001 | included |
| 12 | Emergency status | 0.017 | 0.502 | excluded |
| 13 | Referred to on separation | <0.001 | 0.751 | excluded |
| 14 | Source of referral | 0.003 | 0.018 | included |
| 15 | Complications and comorbidities | <0.001 | 0.002 | included |
| 16 | Alcohol-related primary diagnosis | <0.001 | 0.002 | included |
| 17 | Primary specialty code | <0.001 | 0.300 | excluded |
| 18 | ED visit | <0.001 | <0.001 | included |
| 19 | D&A Service interaction | <0.001 | <0.001 | included |
| 20 | Certain infectious and parasitic diseases | <0.001 | 0.189 | excluded |
| 21 | Neoplasms | <0.001 | 0.799 | excluded |
| 22 | Diseases of the blood and blood-forming organs and certain disorders involving the immune mechanism | 0.058 | excluded | excluded |
| 23 | Endocrine, nutritional and metabolic diseases | <0.001 | 0.362 | excluded |
| 24 | Mental, Behavioural and Neurodevelopmental disorders | 0.048 | 0.599 | excluded |
| 25 | Diseases of the nervous system | 0.698 | excluded | excluded |
| 26 | Diseases of the eye and adnexa | 0.233 | excluded | excluded |
| 27 | Diseases of the circulatory system | 0.010 | 0.670 | excluded |
| 28 | Diseases of the respiratory system | <0.001 | 0.477 | excluded |
| 29 | Diseases of the digestive system | 0.452 | excluded | excluded |
| 30 | Diseases of the skin and subcutaneous tissue | 0.194 | excluded | excluded |
| 31 | Diseases of the musculoskeletal system and connective tissue | 0.021 | 0.866 | excluded |
| 32 | Diseases of the genitourinary system | 0.001 | 0.506 | excluded |
| 33 | Symptoms, signs and abnormal clinical and laboratory findings, not elsewhere classified | <0.001 | 0.177 | excluded |
| 34 | Injury, poisoning and certain other consequences of external causes | 0.572 | excluded | excluded |
| 35 | Codes for special purposes | 0.224 | excluded | excluded |
| 36 | External causes of morbidity | 0.157 | excluded | excluded |
| 37 | Factors influencing health status and contact with health services | 0.348 | excluded | excluded |
| 38 | Length of stay in index admission | 0.002 | 0.050 | excluded |
| 39 | Days in psych unit | 0.353 | excluded | excluded |
| 40 | Hours on mech vent num | 0.364 | excluded | excluded |
| 41 | Number of specialties | 0.543 | excluded | excluded |
| 42 | Number of diagnoses | <0.001 | 0.068 | excluded |

**Appendix Table III. Hospital identifier and region.**

| **Hospital identifier** | **Region** | **Number of admissions** |
| --- | --- | --- |
| P208 | Illawarra | 1,450 |
| P211 | Illawarra | 183 |
| P202 | Illawarra | 44 |
| P206 | Illawarra | 22 |
| P203 | Illawarra | 12 |
| P204 | Illawarra | 9 |
| P207 | Shoalhaven | 442 |
| P205 | Shoalhaven | 70 |
| P291 | Shoalhaven | 22 |
| **Total** | | **2,254** |

**Appendix Table Ⅳ. Receiver operating characteristic curve of models**

| **Model** | **10 independent variables** | **42 independent variables** |
| --- | --- | --- |
| **LR\RF\SVM** | 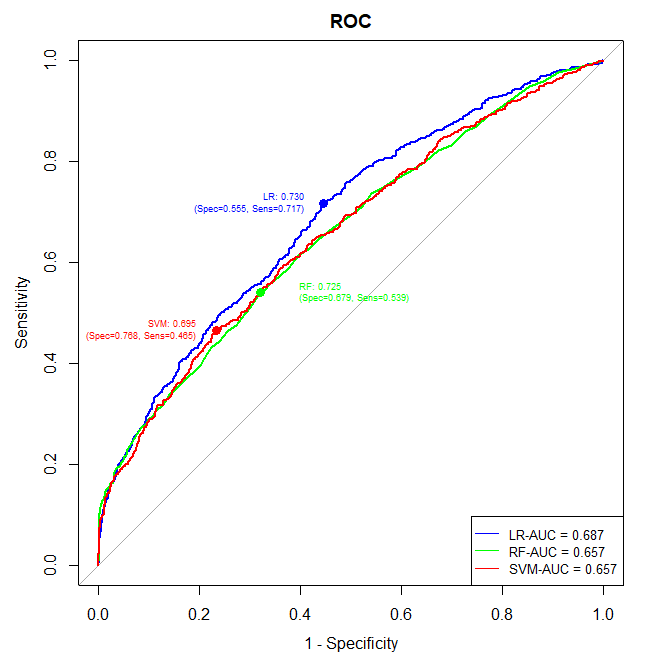 | 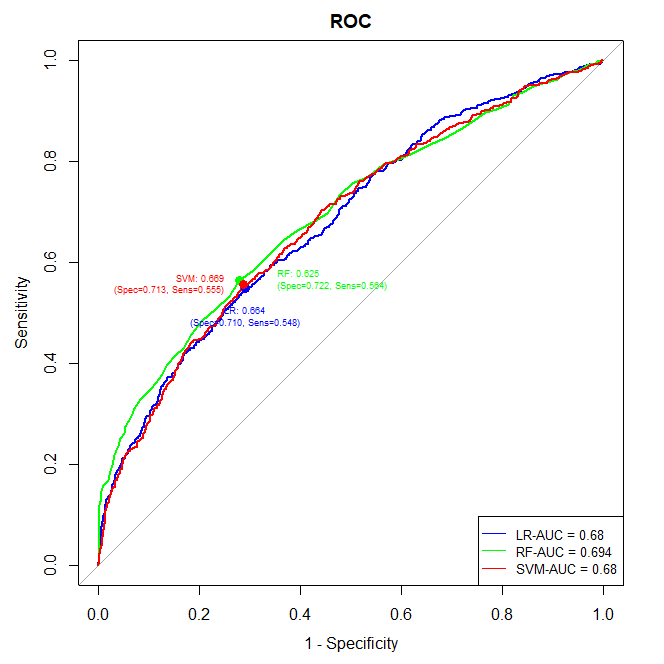 |
| **LSTM** | 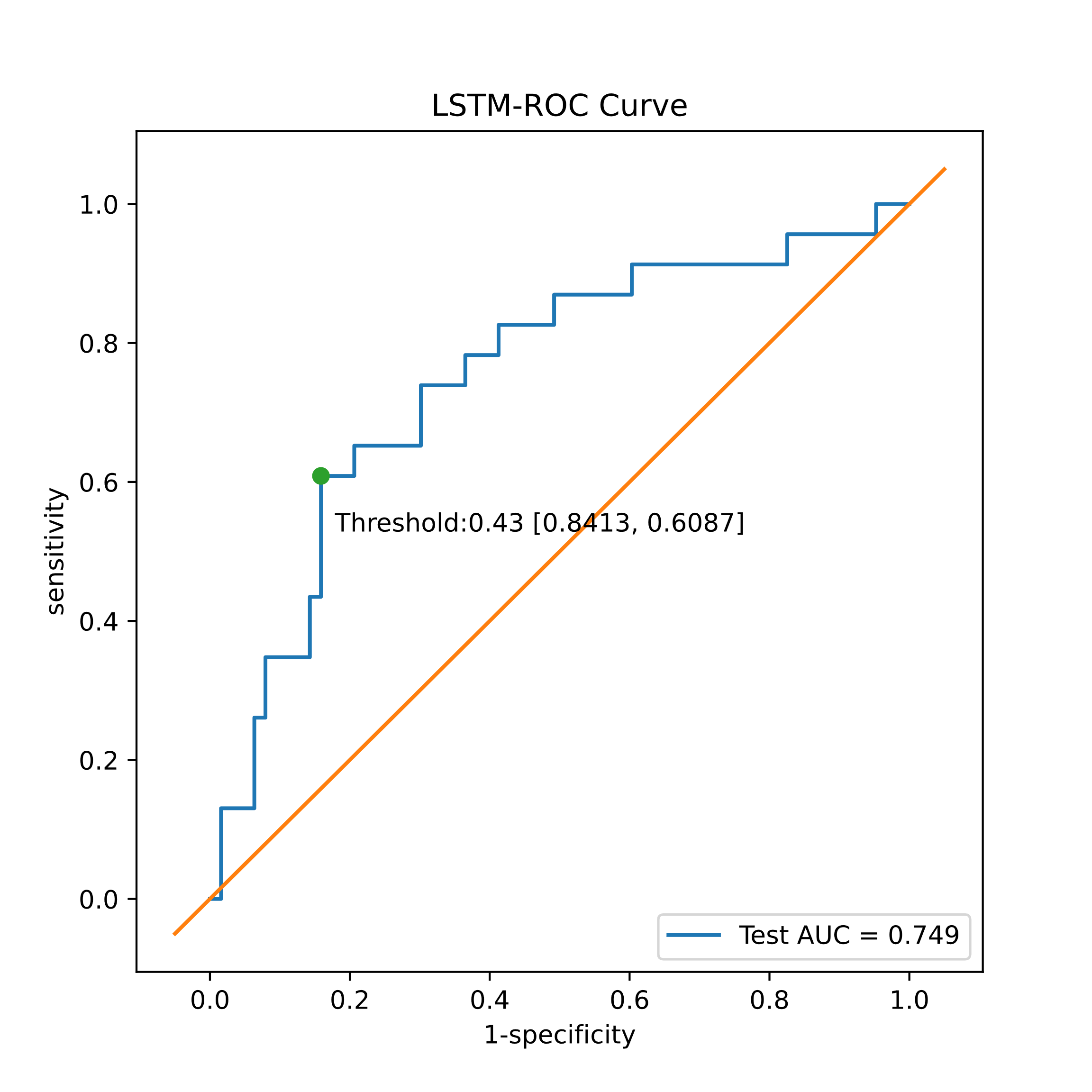 | 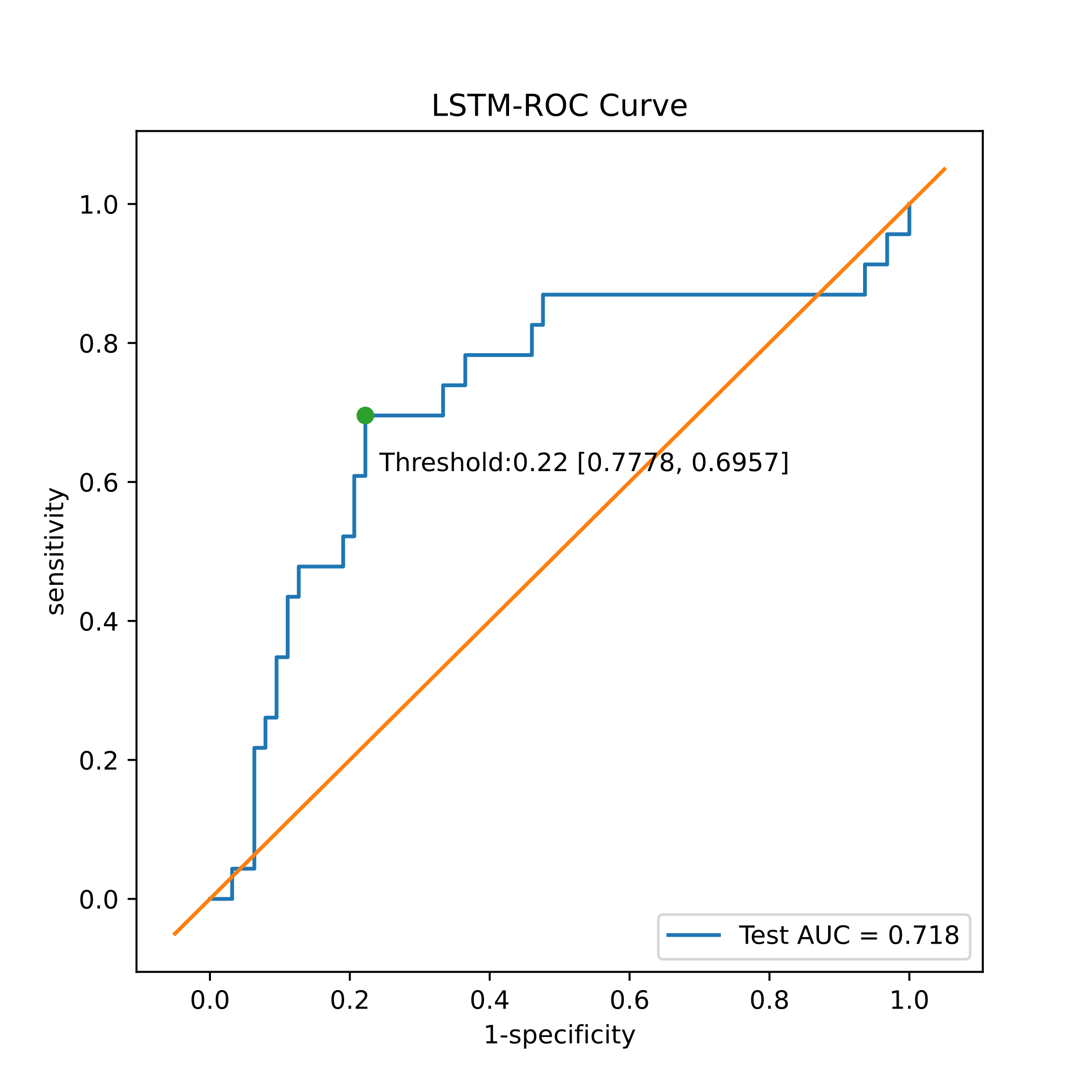 |
| **Clinical Bio-BERT** | 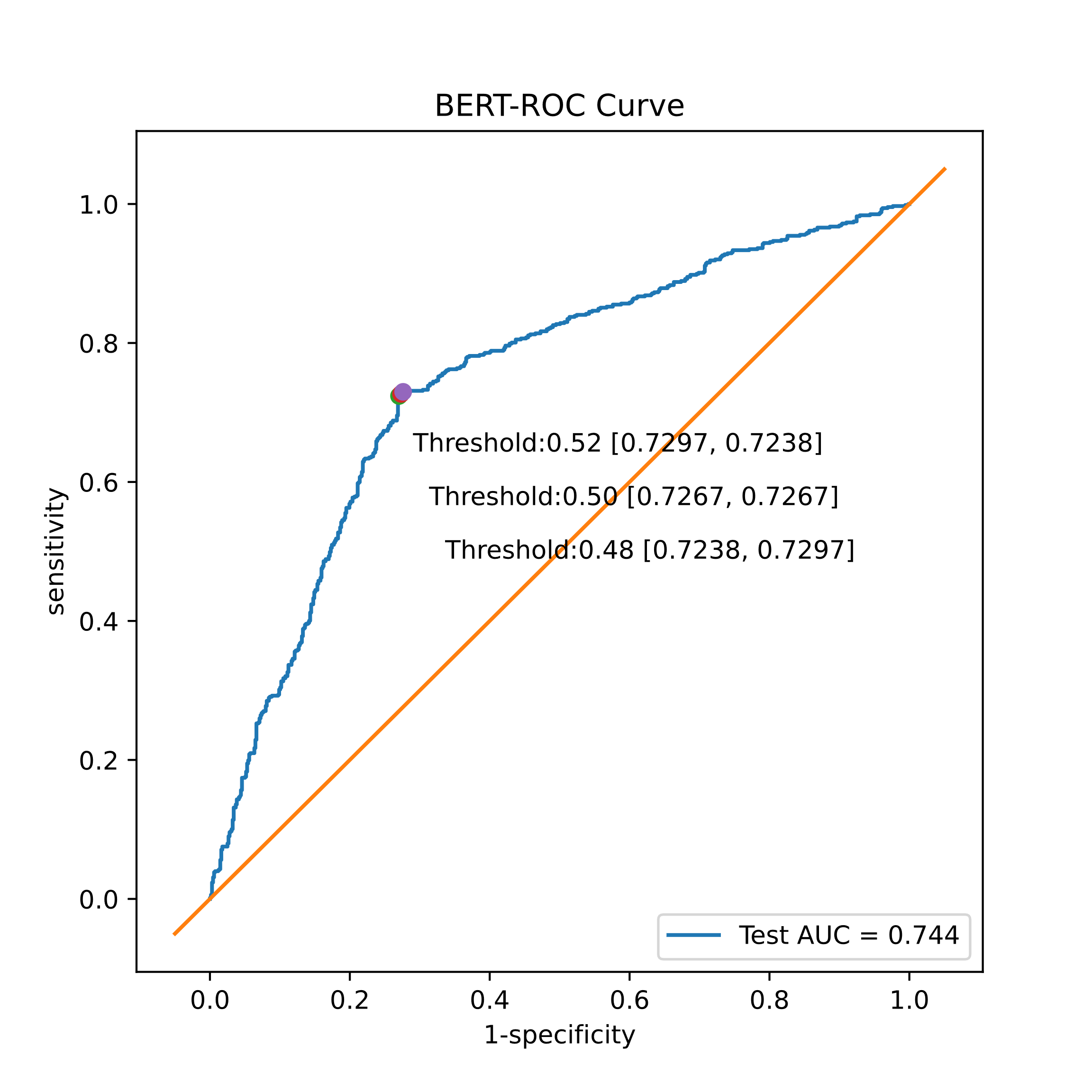 | 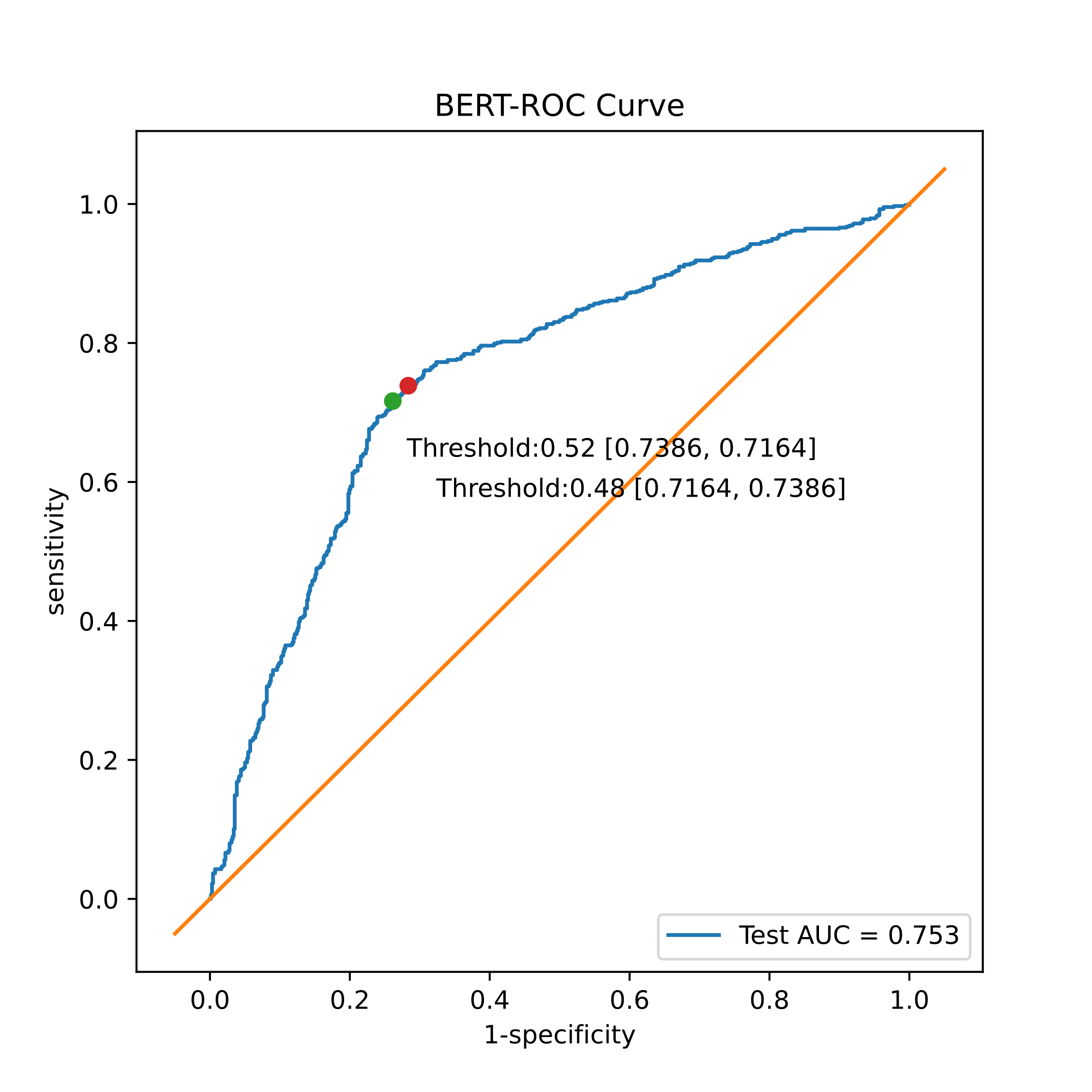 |
